# Comparing Soil Organic Carbon Dynamics in Perennial Grasses and Shrubs in a Saline-Alkaline Arid Region, Northwestern China

**DOI:** 10.1371/journal.pone.0042927

**Published:** 2012-08-10

**Authors:** Yong Zhou, Zhiqin Pei, Jiaqi Su, Jingli Zhang, Yuanrun Zheng, Jian Ni, Chunwang Xiao, Renzhong Wang

**Affiliations:** 1 State Key Laboratory of Vegetation and Environmental Change, Institute of Botany, The Chinese Academy of Sciences, Beijing, China; 2 Graduate School of Chinese Academy of Sciences, Beijing, China; DOE Pacific Northwest National Laboratory, United States of America

## Abstract

**Background:**

Although semi-arid and arid regions account for about 40% of terrestrial surface of the Earth and contain approximately 10% of the global soil organic carbon stock, our understanding of soil organic carbon dynamics in these regions is limited.

**Methodology/Principal Findings:**

A field experiment was conducted to compare soil organic carbon dynamics between a perennial grass community dominated by *Cleistogenes squarrosa* and an adjacent shrub community co-dominated by *Reaumuria soongorica* and *Haloxylon ammodendron*, two typical plant life forms in arid ecosystems of saline-alkaline arid regions in northwestern China during the growing season 2010. We found that both fine root biomass and necromass in two life forms varied greatly during the growing season. Annual fine root production in the perennial grasses was 45.6% significantly higher than in the shrubs, and fine root turnover rates were 2.52 and 2.17 yr^−1^ for the perennial grasses and the shrubs, respectively. Floor mass was significantly higher in the perennial grasses than in the shrubs due to the decomposition rate of leaf litter in the perennial grasses was 61.8% lower than in the shrubs even though no significance was detected in litterfall production. Soil microbial biomass and activity demonstrated a strong seasonal variation with larger values in May and September and minimum values in the dry month of July. Observed higher soil organic carbon stocks in the perennial grasses (1.32 Kg C m^−2^) than in the shrubs (1.12 Kg C m^−2^) might be attributed to both greater inputs of poor quality litter that is relatively resistant to decay and the lower ability of microorganism to decompose these organic matter.

**Conclusions/Significance:**

Our results suggest that the perennial grasses might accumulate more soil organic carbon with time than the shrubs because of larger amounts of inputs from litter and slower return of carbon through decomposition.

## Introduction

Soil carbon pool, as the major part of the terrestrial carbon reservoir, plays a critical, yet poorly understood role in the global carbon cycle [1]. Consequently, the study of soil carbon dynamics is crucial to our ability to understand the global carbon cycle and its response to future global change [2]. Semi-arid and arid regions, which are particularly sensitive to global change, account for about 40% of the terrestrial surface of Earth [3], [4] and contain approximately 10% of the global soil organic carbon stock [5]. A slight change of these carbon pools could have significant impacts on regional or global carbon cycle. Although semi-arid and arid regions are important source and sinks of carbon [4], there is no consistent conclusion on the net carbon effect of semi-arid and arid regions on carbon sequestration partially because of the unique characteristics of arid ecosystems (e.g. exceptional vulnerability, spatial heterogeneity) and the complex responses of biogeochemical cycling to a range of environmental conditions, such as water availability, precipitation pulse, solar radiation, or even heat and wind [6].

The difference between inputs from primary production and the return of carbon to atmosphere through decomposition of organic matter determines the soil carbon budget in semi-arid and arid ecosystems, and other terrestrial ecosystems [3]. Fine root production contributes to about 33% of annual net primary productivity and its turnover directly impacts the biogeochemical cycle and sequestration of carbon [7], [8]. Therefore, precise estimates of fine root production and turnover rate are essential for a meaningful evaluation of soil organic carbon budgets in semi-arid and arid regions. However, the vast majority of previous studies have placed more emphasis on fine root growth and distribution which were closely related to exploit soil resources, such as soil water and nutrient in semi-arid and arid regions [9]–[11]. Although some studies have tried to address fine root biomass and dynamics in arid regions [11], [12], few data on the contribution of fine roots to soil carbon cycle have been reported from saline-alkali arid regions.

Litterfall and its decomposition are critical steps in forming soil organic matter, mineralizing organic nutrients, thus balancing soil carbon in terrestrial ecosystems [13]. It is well established that litter production is largely determined by soil water availability, soil fertility, species composition, plant density in arid ecosystems [14], [15] and litter decomposition depends on a variety of factors such as litter quality, precipitation, soil biotic activity, soil resource availability and solar radiation [3], [6], [13], [16], [17]. While a number of recent studies have described the ecological role of litterfall in soil carbon cycling for semi-arid and arid communities in different parts of the world [14], [15], [18], [19], its role has not yet been described for saline-alkali communities in central Asia.

In arid ecosystems, perennial grass and shrub are two typical plant life forms. Selective grazing leads to the reduction of plant coverage, or even induces the replacement of plant life forms from herbaceous plants, mostly perennial grasses, to long-lived woody shrubs. Different plant life forms forming different micro-environment and providing different quantity and quality of litter may play different in nutrient cycling and ecosystem functioning within the same climatic region [20]. Generally, perennial grasses with show-root systems would be more likely to use erratic and discontinuous water sources from the upper soil layers while shrubs with deep-root systems would use more stable water resources in deeper soil layer [9], [21], [22]. Perennial grasses and shrubs also differ in litterfall chemistry and in the quantity and timing of litterfall [20], [23], which directly affect decomposition processes, nutrient cycling and other related ecosystem processes [24]. Further, changes in the quality and/or quantity of litter (including dead root) inputs might shift the properties of soil microorganism like microbial biomass, activity.

Several studies have separately compared the ecological roles of fine root or litterfall between perennial grasses and shrubs [11], [20], [23], [24] but studies addressing simultaneously the effects of fine root, litterfall and their decomposition on soil organic carbon dynamics between perennial grasses and shrubs in saline-alkali arid region are relatively scarce. In this study, our overall aim is to compare soil organic carbon dynamics between adjacent perennial grasses and shrubs in a saline-alkali arid region in northwestern China. Furthermore, our specific objectives were to examine: (1) How would the dynamics of fine root mass, litterfall and floor mass differ between the perennial grasses and the shrubs? (2) Would differences in site conditions, soil microbial properties and litter chemistry affect leaf litter decomposition rate thus result in a difference in SOC pool between the perennial grasses and the shrubs?

## Results

### Soil Water Content

**Figure 1:**
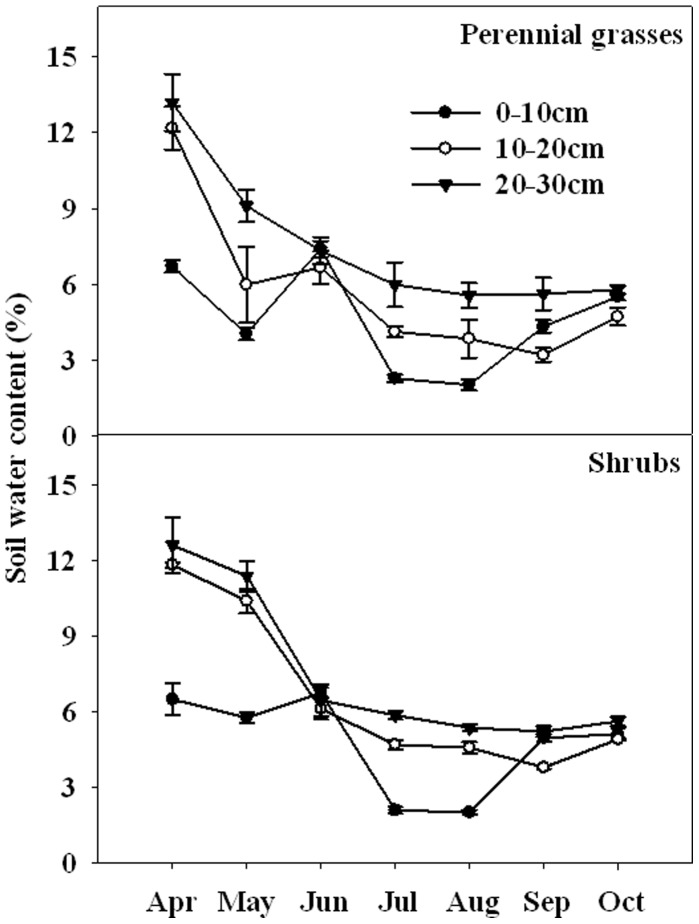
Seasonal variations of gravimetric soil water content (SWC) in the perennial grasses (a) and the shrubs (b) from April to October 2010 for soil layers 0–10 and 10–20, 20–30 cm. Vertical bars indicate standard errors of means (n = 3).

Gravimetric soil water content (SWC) differed significantly between life forms in the growing season 2010 (*P*<0.05, Table 1). Integrated over all depths and averaged over the growing season, SWC in the perennial grasses (6.0%) was 5.0% lower than the shrubs (6.3%) (Fig. 1). SWC significantly increased with soil depth (*P*<0.001) and varied greatly among sample dates (*P*<0.001, Table 1).

**Table 1:** Results (F-values) of repeat-measurement ANOVA on the effects of life form (LF), soil layer (SL), sampling date (SD), and their interactions on soil water content (SWC), soil organic carbon (SOC), fine root biomass (FRB), necromass (FRN), also the effects of life form (LF), sampling date (SD) and their interactions on floor mass (FM), aboveground litterfall (AL), soil microbial biomass carbon (SMBC), soil microbial activity (SMA) and *q*CO_2_.

Source	SWC	SOC	FRB	FRN	AL	FM	SMBC	SMA	*q*CO_2_
LF	4.90*	28.04***	36.49***	2.07	0.70	16.44*	4.09	8.39*	12.48*
SD	123.90***	1.11	6.08**	10.10***	3.32*	1.99	73.39***	149.99***	3.08
LF×SD	7.19***	1.95	5.79**	7.33***	3.50*	0.81	0.06	11.90**	2.84
SL	134.97***	63.17***	49.02***	10.26**					
LF×SL	3.15	1.40	6.36***	0.17					
SD×SL	15.21***	0.49	3.46*	4.30***					
LF×SD×SL	0.49	0.37	1.96	2.64*					

* , **, and *** represent significant at P<0.05, 0.01, and 0.001, respectively.

### Fine Root Biomass, Production and Turnover Rate

**Figure 2:**
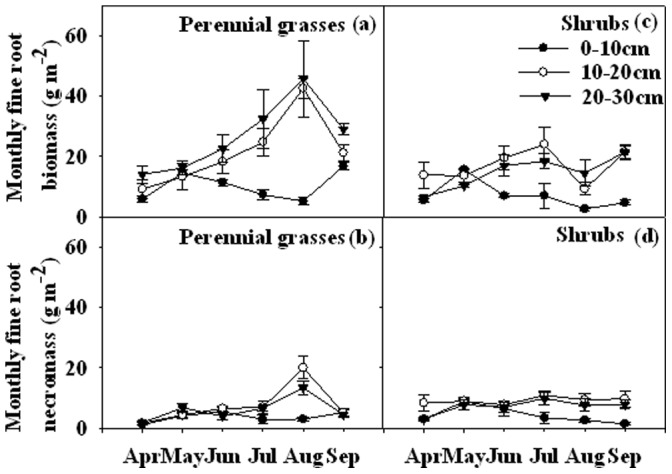
Seasonal variations of fine root biomass (a, c) and necromass (b, d) in the perennial grasses (a, b) and the shrubs (c, d) from April to September 2010 for soil layers 0–10, 10–20, and 20–30 cm. Vertical bars indicate standard errors of means (n = 3).

Integrated over all depths and averaged over the growing season, mean fine root biomass in the perennial grasses (58.4 g m^−2^) was 33.6% significantly higher than the shrubs (38.8 g m^−2^) in 2010 (*P*<0.001, Table 1, Fig. 2a, c). The vertical distribution of fine root biomass significantly increased with soil depths in the perennial grasses, while in the shrubs fine root biomass was highest in 10–20 cm of the soil profile in the growing season (Fig. 2a, c). Seasonal variations of fine root biomass showed different patterns between the perennial grasses and the shrubs (Fig. 2a, c).

Fine root necromass did not differ significantly between the perennial grasses (17.1 g m^−2^) and the shrubs (20.5 g m^−2^) in 2010 (Table 1, Fig. 2b, d), but showed significant differences between soil layers (*P*<0.01, Table 1, Fig. 2b, d). Significant differences among sampling dates on fine root necromass were observed in the growing season of 2010 (*P*<0.001, Table 1).

**Figure 3:**
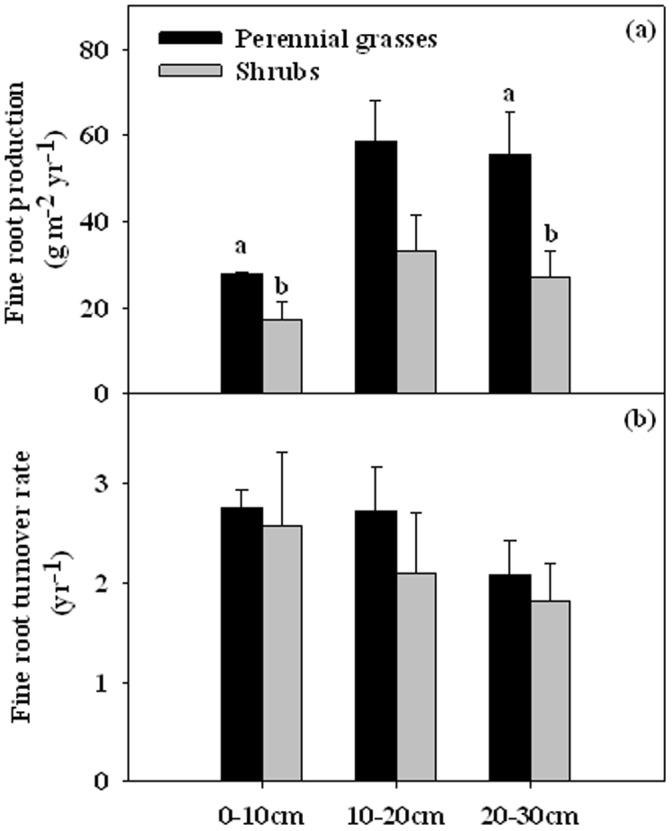
Fine root production (a) and fine root turnover rate (b) in the perennial grasses and the shrubs for soil layers 0–10, 10–20 and 20–30 cm. Vertical bars indicate standard errors of means (n = 3). Difference letters indicate statistically significant differences (P<0.05), and absence of letters implies that no significant differences were detected.

Fine root production was significantly differed between life forms (*P*<0.001). Integrated over all depths, fine root production in the perennial grasses (142.2 g m^−2^ yr^−1^) was 45.6% higher than the shrubs (77.4 g m^−2^ yr^−1^) (Fig. 3a). Fine root turnover rate did not significantly differ between the perennial grasses (2.52 yr^−1^) and the shrubs (2.17 yr^−1^) (Fig. 3b).

### Aboveground Litterfall and Floor Mass

**Figure 4:**
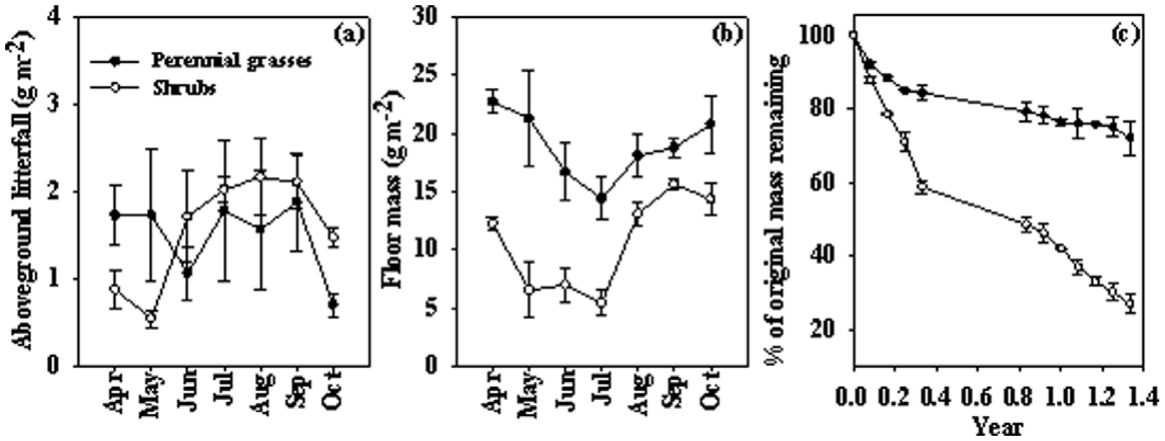
Seasonal variations of aboveground litterfall (a), floor mass (b) in the perennial grasses and the shrubs from April to October 2010 at monthly intervals. Litterfall mass remained (% of original) (c) in the perennial grasses and the shrubs during more than one year period. Vertical bars indicate standard errors of means (n = 3).

No significant differences were found between the perennial grasses (10.6 g m^−2^ yr^−1^) and the shrubs (10.9 g m^−2^ yr^−1^) in litterfall in the growing season 2010 (Table 1, Fig. 4a), whereas the effects of sampling date were statistically significant for litterfall (*P*<0.05, Table 1). The seasonal pattern of litterfall in the perennial grasses exhibited bimodal peaks separately in the beginning and the drought of the growing season while the shrubs showed a clear uni-modal pattern in dry season (Fig. 4a).

Mean floor mass in the perennial grasses (19.0 g m^−2^) was 44.2% significantly higher than the shrubs (10.6 g m^−2^) in the growing season 2010 (*P*<0.05,Table 1, Fig. 4b). The seasonal patterns of floor mass were very similar between the perennial grasses and the shrubs, with the largest floor mass in August to October, and to next year’s April (Fig. 4b).

### Leaf Litter Decomposition

Leaf litter decomposition rate was significantly different between the perennial grasses and the shrubs (Table 2, Fig. 4c). The mass loss of the perennial grasses leaves (20.9% yr^−1^, in comparison with its original mass) was significantly lower than the shrubs leaves (54.9% yr^−1^) (Table 2). The mass loss rate declined with incubation time. Significant difference in mass loss rate between bags was detected from the first three months and the first ten months of the incubation period for the perennial grasses and the shrubs, respectively. The decay constant, *k* (mean ± SE), of the perennial grasses was 0.19 and of the shrubs was 0.84 (Table 2).

**Table 2:** Mean annual decay constant (k) of aboveground litterfall in the perennial grasses and the shrubs.

Life form	*k*	R^2^	P	Mass loss % yr^−1^
Perennial grasses	0.19(0.00)b	0.89	<0.0001	20.9(3.5)b
Shrubs	0.84(0.03)a	0.96	<0.0001	54.9(1.9)a

Values in the parentheses indicate standard error (n = 3). Difference letters indicate statistically significant differences (P<0.05).

### Soil Microbial Properties

**Figure 5:**
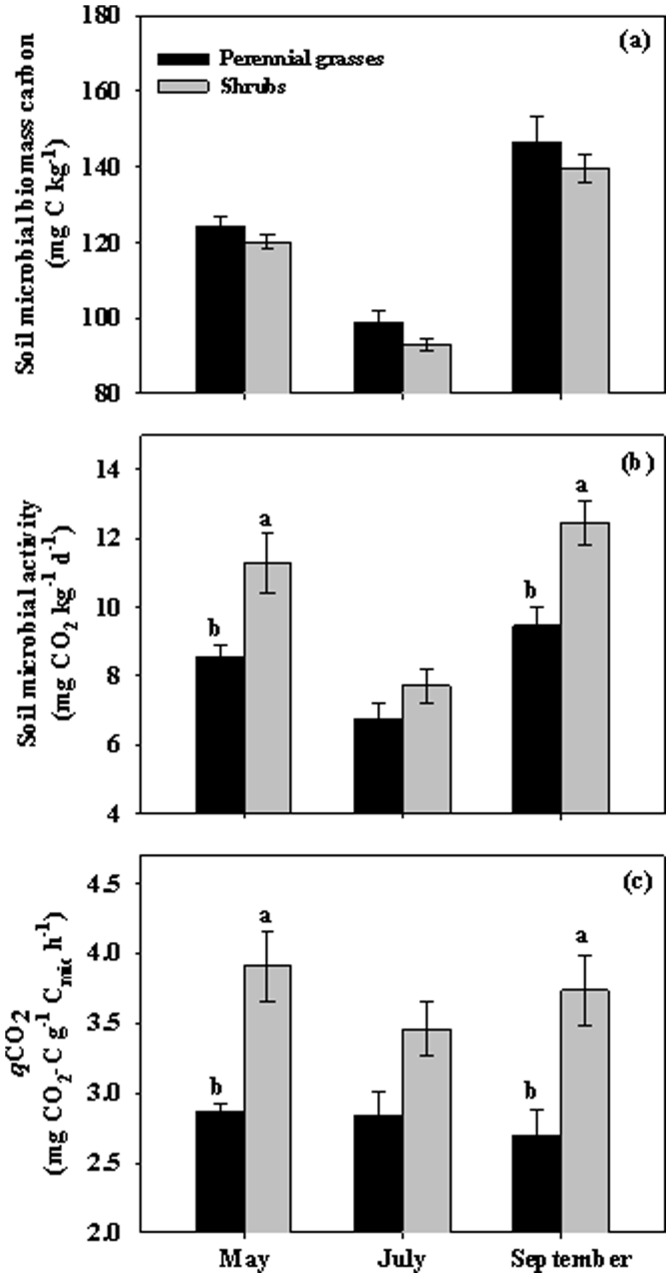
Soil microbial biomass carbon (a), soil microbial activity (b) and *q*CO_2_ (c) in the perennial grasses and the shrubs in May, July and September in 2010. Vertical bars indicate standard errors of means (n = 3). Difference letters indicate statistically significant differences (P<0.05), and absence of letters implies that no significant differences were detected.

Although Soil microbial biomass carbon (SMBC) did not significantly differ between two life forms, Soil microbial activity (SMA) was significantly lower in the perennial grasses than in the shrubs (*P*<0.05, Table 1). More specifically, SMA in the perennial grasses was 31.7%, 14.6% and 31.8% lower than in the shrubs among the three measured months (Fig. 5b). Meanwhile, in both life forms, both SMBC and SMA were significantly higher in May and September than July 2010 (*P*<0.001, Table 1, Fig. 5a, b). *q*CO_2_ as a measure of microbial efficiency was significantly lower in the perennial grasses than in the shrubs but showed no variation among the three measured months (*P*<0.05, Table 1, Fig. 5c).

### Soil Organic Stocks and Inputs

Total SOC was significantly different between two life forms (*P*<0.001, Table 1). Integrated over all depths, mean total SOC in the perennial grasses (1.32 Kg C m^−2^) was 15.2% higher than in the shrubs (1.12 Kg C m^−2^) in 2010. SOC significantly decreased with soil depth (*P*<0.001, Table 1). Soil organic inputs were calculated by multiplying production (fine root production and leaf litter production) to carbon content. Total fine root carbon input in the perennial grasses (43.2 C m^−2^ yr^−1^) was 27.8% higher than the shrubs (31.2 g C m^−2^ yr^−1^). No significant difference was detected in litterfall carbon input between the perennial grasses and the shrubs.

## Discussion

### Fine Root Dynamics

Seasonal dynamics of fine root biomass and necromass for perennial grasses and shrubs have been reported in different parts of semi-arid and arid regions [12], [25]. However, no consistent patterns have been observed. In our study, fine roots biomass in both the perennial grasses and the shrubs at the top soil (0–10 cm) fleetingly increased in spring, then gradually decreased and reached the lowest at the end of August, then slightly increased in September (Fig. 2a, c). This might be attributed to low and continuous decrease of soil water content (Fig. 1) at the top soil and increase of temperature that limited soil water availability. In contrast, the patterns of fine roots biomass in the perennial grasses and the shrubs are similar at 10–20, 20–30 cm, continuously increase from April to July, the only difference is that in the perennial grasses fine roots biomass reached the peak while the shrubs sharply decreased at the end of August (Fig. 2a, c).

This difference might largely be explained by the different responses to water stress between the perennial grasses and the shrubs. It is well established that perennial grasses with shallow-root systems would use more discontinuous and erratic water sources in the upper soil layers while shrubs with deep-root systems would use more stable water in deeper soil layers [9], [21], [22]. However, in our study, both *R. soongorica* and *H. ammodendron* belonged to other root types instead of deep root type [26], [27]. Xu and Li [26] reported that both *R. soongorica* and *H. ammodendron* depended on water in shallow upper soil and when long time lack of effective precipitation generated extreme drought in upper soil, both *R. soongorica* and *H. ammodendron* could shrink their roots in upper soil to effectively adjust the ratio of roots absorption area to canopy assimilation area so as to maintain regular carbon uptake [26], [27].

Therefore, the observed decrease of fine root biomass in the shrubs might be attributed to the strategies of *R. soongorica* and *H. ammodendron* to water stress after a long lack of rainfall in August. Moreover, our estimates of mean fine root biomass revealed that fine root biomass of the perennial grasses was more abundant than the shrubs near the upper soil layers, implying that the former were better competitors for water in spatial dimension, particularly for pulsed rainfall.

As for fine root necromass, we observed bimodal peaks separately in the beginning and near the end of the growing season in the perennial grasses while in shrubs the necromass slightly increased in the beginning of the growing season and did not obviously change in the remaining (Fig. 2b, d). Fine root necromass in the perennial grasses slightly increased in the beginning of the growing season might be linked to senescence of some portions of the roots after the winter while the steep increase at the end of the growing seasons was followed by the peak of fine root biomass.

Fine root production and turnover are important components of carbon budget of plants and key regulators of nutrient cycles of ecosystems. However, due largely to technical limitations, accurate measurement of fine root production is hindered by estimating methods which are based on potentially tenuous assumptions and are subject to sampling errors [28]. In our study, we chose the Decision matrix method [29] to estimate fine root production. Fine root production in the perennial grasses (142.2 g m^−2^ yr^−1^) was greater than in shrubs (77.4 g m^−2^ yr^−1^) (Fig. 3a). Except for the nature of species, another possibly reason for high fine root production in the perennial grasses compared to the shrubs is the relatively favorable microclimatic conditions.

Fine root turnover rate varies within and among species and across ecosystems, also estimates vary depending on the method used. Although our fine root turnover rates (2.54 and 2.17 yr^−1^ for the perennial grasses and the shrubs, respectively) are larger than those reviewed by Gill and Jackson [8] for both grassland and shrubland, they are comparable to the values reported in a semi-arid temperate steppe (2.11∼2.23 yr-1) [25] and in Mojave Desert shrub communities (2.33∼2.99 yr-1) [30]. Moreover, the result that fine root turnover rate in perennial grasses was higher than the shrubs was similar to that reported by Gill and Jackson [8].

### Soil Microbial Properties

Soil microbial biomass (SMB) represents a small but important labile pool of nutrients in soils, and its activity exerts a key controlling influence on the rate which C, N and other nutrients cycle through ecosystems [31]. Although not significant, slightly higher SMB-C in the perennial grasses than in the shrubs in our study might be linked to higher soil organic content in the perennial grasses. Higher SOC concomitant with higher SMB-C was also found in other studies [32]. Significantly higher SMA in the shrubs than in the perennial grasses might be attributed to higher nutrient availability in soil [33]. Our leaf litter bag experiment suggested that the perennial grasses produced more poor quality of organic matter than the shrubs. Similar patterns of seasonal variations (with higher values in May and September than in July) of SMB-C and SMA were also observed in other studies [34], [35]. Griffiths et al. [36] reported that water availability affects the physiological status of bacteria and can indirectly regulate substrate availability and water stress may reduce microbial biomass and activity through induced osmotic stress, resource competing and starvation [33], [36]. In our study, soil water content at 0–10 cm was extreme low in July both in the perennial grasses and in the shrubs and monthly mean air temperature reached the peak of that year. These extreme drought conditions might restrain the amounts of microbial biomass and their activity. The sharp decline observed in drought season partly suggested that the microclimate conditions, especially the soil moisture, predominate over the availability of substrates in regulating seasonal variability of microbial biomass production and activity at 0–10 cm soil layer in arid ecosystems. The lower microbial efficiency (*q*CO_2_) for the perennial grasses might be ascribed to lower quality of organic matter inputs derived from perennial grasses [32].

### Leaf Litter Dynamics

Previous studies have indicated that litterfall could show no distinct seasonal pattern, uni-modal, bimodal, or even multi-modal peaks [12], [15]. In our study, litterfall of the perennial grasses exhibited bimodal peaks while the shrubs showed a clear uni-modal pattern. Seasonal patterns of litterfall are largely attributed to inherent nature of the species and associated factors responsible for senescence and abscission. The first peak of litterfall production in the perennial grasses could be linked to the abscission of older generations of dead leaves (standing litter) when new leaves gradually appeared in the beginning of the growing season. Moreover, dry seasonal litterfall peaks observed in our study have been reported in several litterfall studies [12], [15], [37], [38]. In arid ecosystems, water stress which is usually extreme in the dry season might be an important precipitating factor influencing dry season leaf fall [15]. Moore [39] reported that water stress could initiate the synthesis of abscisic acid, which can stimulate the senescence of leaves and other plant parts. Zhou et al. [40] reported that air temperature (especially maximum and effective temperature) was an important environmental variable in affecting litterfall production. In our study, from July to September, rare rainfall concomitant with high air temperature induced a peak of litterfall production in the perennial grasses and the shrubs.

Annual litterfall production values were similar between the perennial grasses (10.6 m^−2^ yr^−1^) and the shrubs (10.9 m^−2^ yr^−1^) in our study, which were much lower than those reported in other semi-arid and arid ecosystems [12], [14], [15], [18], [19] with different species, canopy development and coverage, soil and weather conditions. The low number of samples in our study might underestimate the annual litterfall production. However, we do not expect these biases will change our conclusion on the comparison of litterfall production between the perennial grasses and the shrubs. Nonetheless, our litterfall production was similar to the value of 11.6 g m^−2^ yr^−1^ reported by Weatherly et al. [41] in a Mojave Desert ecosystem. Meanwhile, floor mass on the soil surface serves as temporary sink for nutrients which are released gradually through decomposition, thereby providing a permanent source of nutrients to the soil [15]. In the present study, the perennial grasses had more floor mass than the shrubs, possibly because the former had a lower decomposition rate.

Although lack of directly comparable data of leaf litter decomposition rates for *C.squarrosa*, *R. soongorica* and *H. ammodendron*, the percentage of 1-year weight loss from the perennial grasses (20.9%) in our study was comparable to the rate for a perennial grass community in the Patagonian steppe (31% after 20 months) [17]. And annual decomposition rates of the shrubs (54.9%), expressed in percentage of original mass, also fell within the range rates reported for shrubs in other arid regions (35%–70% for a Northern American desert community [42]; 40% for a Chihuahuan Desert community [43]; 35%–40% for a Mojave Desert community [41]). Rapid initial decomposition and slow latter decomposition (Fig. 4c) observed in our study could be likely linked to rapid loss of labile compounds in leaf litter. Meanwhile, higher leaf litter decomposition rate for shrubs can be ascribed to the following reasons. First, Cornwell et al. [44] reviewed that plant species traits are predominant control on litter decomposition rates. Globally, herbaceous species in general did not produce litter that decomposed faster than woody species and eudicot litter decomposed on average 1.6 times faster than monocot litter [44]. Correspondingly, in our study, Leaf litter decomposition rates were significantly higher in the shrubs (both *R. soongorica* and *H. ammodendron* are dicotyledon) than in the perennial grasses (*C.squarrosa* is monocotyledon). Besides, litter chemistry, particularly the concentration of nutrient and carbon compounds, is a major control on litter decomposition [13]. C: N ratio has been identified as key features determining the rate of litter decomposition, and litter with high C: N ratio decompose at relatively low rates [44], [45]. In the present study, we also detected that the C: N ratio of the perennial grasses (47.8) was significantly higher than the shrubs (17.2). In addition, higher soil microbial activity in the shrubs observed in our study indirectly suggested that higher ability of microorganism to decompose leaf litter from the shrubs.

### Soil Organic Carbon Stocks and Inputs

Soil organic carbon storage, which represents the accumulated difference between inputs from primary production and outputs through decomposition, is critical to estimate effective carbon sequestration capacity of ecosystems. In our study, the observed total soil organic carbon stocks (1.32 and 1.12 Kg m^−2^ for the perennial grasses and the shrubs, respectively) are within the range reported by Feng et al. [46] for 17 locations in different types of desert in China (0.02–4.97 kg m^−2^ for 0–20 cm profile). Total soil carbon stocks were higher in the perennial grasses than in the shrubs. This accumulative discrepancy might ascribe to the following two main reasons. First, soil organic carbon inputs through primary production (here is the sum of fine root production and litterfall) in the perennial grasses were higher than in the shrubs. Second, our litter bag experiment and measured soil microbial properties suggested that inputs from the perennial grasses are in poor quality and relatively resistant to decay by lower ability of microorganism. Therefore, our results suggested that the perennial grasses might accumulate more soil organic carbon with time than the shrubs because larger amounts of inputs from litter and slower return of carbon through decomposition in the perennial grasses than in the shrubs.

### Conclusions

Our results show that there are obvious differences in fine root biomass, production, turnover, floor mass, leaf litter decomposition and soil microbial properties between the perennial grasses and the shrubs. The absolute size of soil organic carbon stocks increased with time in the perennial grasses is likely due to greater inputs from litter and slower outputs through decomposition. Our findings provide sound and basic knowledge in estimating the effectiveness of soil organic carbon dynamics and sequestration in the two type of life forms in saline-alkali arid regions of China and might be useful to address the eventual effects of plant species shifting on ecosystem functioning. Nonetheless, the low number of samples taken in our study might not accurately reflect the differences between the perennial grasses and the shrubs in soil carbon dynamics due to the high heterogeneity in arid ecosystems. Also, our study only conducted one year, and annual rainfall during that year was higher than the long-term mean values. Ecological processes in water-limited ecosystems might vary sharply with water availability. These remarks should be taken into consideration in the future studies.

## Materials and Methods

### Ethics Statement

Fukang Desert Ecosystem Research Station is a department of Xinjiang Institute of Ecology and Geography, Chinese Academy of Sciences. This study was approved by State Key Laboratory of Vegetation and Environmental Change, Institute of Botany, the Chinese Academy of Sciences and Fukang Desert Ecosystem Research Station.

### Site Description

This study was conducted during the growing season of 2010 in the vicinity of the Fukang Desert Ecosystem Research Station (44°17′N, 88°56′E) of the Chinese Academy of Sciences, which is located at a transitional area of oasis-desert and the southern edge of the Gurbantonggut Desert. This region has a typical continental arid temperate climate, with a long-term mean annual precipitation <150 mm but a potential mean annual evaporation >2000 mm, and has a long cold winter and hot summer, with a long-term mean annual temperature of 4–6°C. Soils at this region are characterized as Yermi-sodi-Haplic Luvisol [47]. Physical properties of the soils in the perennial grasses and the shrubs sites in layers of 0–10, 10–20 and 20–30 cm are shown in Table 3.

**Table 3:** Soil pH, bulk density and texture of this study.

	0–10 cm		10–20 cm		20–30 cm	
	Perennial grasses	Shrubs	Perennial grasses	Shrubs	Perennial grasses	Shrubs
pH	8.46(0.07)	8.60(0.20)	8.55(0.07)	8.59(0.04)	8.72(0.05)	8.85(0.08)
Soil bulk density (g cm^−3^)	1.18(0.03)	1.20(0.00)	1.31(0.02)	1.33 (0.02)	1.43 (0.02)b	1.48(0.02)a
Clay, <2 µm (%)	3.06(0.02)b	3.62(0.04)a	8.12(0.04)	7.97(0.57)	8.16(0.01)a	7.73(0.02)b
Silt, 2–50 µm (%)	31.19(0.22)b	33.47(0.30)a	55.29(0.16)b	59.80(0.68)a	55.58(0.16)	55.16(0.08)
Sand, 50–1000 µm (%)	65.76(0.24)a	62.91(0.33)b	36.59(0.19)a	32.23(0.11)b	36.26(0.16)b	37.10(0.10)a

Values in the parentheses indicate standard error (n = 3). Difference letters indicate statistically significant differences (P<0.05), and absence of letters implies that no significant differences were detected.

We established three plots (each 25 m×25 m) for each plant life form for field measurements during the growing season of 2010. The perennial grasses community is dominated by *Cleistogenes squarrosa* which is widely distributed in the Eurasian steppe zone [48]. *C. squarrosa* is a perennial bunchgrass with characteristic C_4_ anatomy and a fibrous root system [48], and is also considered as a key species for sustainable grassland development [49]. The shrubs community is co-dominated by *Reaumuria soongorica* and *Haloxylon ammodendron*. *R. soongorica*, an extreme xeric semishrub of Tamaricaceae, is the constructive and dominant species of the desert ecosystems in the Central Asia, and forms the vast and distinctive landscape of the salt desert [50]. *H. ammodendron*, a shrubby perennial of Chenopodiaceae with characteristic C4 anatomy, is an important component of old Mediterranean flora [51]. The deserts communities which dominate or co-dominate by *H. ammodendron* are the most widely distributed vegetation types of Asian deserts [52]. All the plots are relatively flat with a slope of <5° and an elevation of about 450 m above sea level. In the perennial grasses sites, mean density, height and ground cover are about 60000 clusters ha^−1^, 0.14 m and 50%, respectively. In the shrubs sites, mean density, height and ground cover of *R. soongorica* are about 2500 stems ha^−1^, 0.34 m and 23%, respectively. For *H. ammodendron* they are about 500 stems ha^−1^, 1.07 m and 10%, respectively.

### Soil Sampling and Soil Properties

Soil samples (0–10, 10–20, 20–30 cm) for soil organic carbon, soil water content, and fine root mass were randomly collected from five cores (8 cm diameter) in each plot per month. Besides, soil samples (0–10 cm) for analyzing microbial properties were done during May, July and September. Fresh samples for determination of soil microbial properties were immediately transported to the laboratory with a portable ice box and stored at 4°C before analysis. In the laboratory, these soil samples for analyzing soil organic carbon, soil physical properties, and soil microbial properties were passed through a 2-mm sieve and manually cleaned off any visible plant tissues.

We calculated soil carbon stock (g m^−2^) for each soil sample at different depths by measuring bulk density (g m^−3^) and mass-based soil carbon content (%). Bulk density of soil was determined by weighing air-dried volume samples. Soil carbon content was measured by the dichromate oxidation method [31]. Briefly, finely grinded 0.2 g soil was digested with 5 ml 2 M K_2_Cr_2_O_7_ and 5 ml of concentrated H_2_SO_4_ at 170°C for 10 min, then titrated the digests with 2 M standardized FeSO_4_.

Gravimetric soil water content (SWC) was measured by oven-drying samples at 105°C for 24 h. Soil pH was measured in the 1∶5 soil/water extract, electrical conductivity in the saturated paste extract (ECsp). The particle size distribution of the <2 mm particle fractions was determined using the laser detection technique.

### Fine Root Mass, Production and Turnover Rate

Fine roots (<2 mm) were manually washed free of soil samples and separated into live and dead root fragments by their color, resilience, consistency and the degree of cohesion between the cortex and the periderm. The separated fine roots were oven-dried to constant mass at 65°C for determination of mass and chemical analysis. Fine root production was estimated using the Decision matrix method [29]. Fine root turnover rate was calculated as the ratio of the total amount of fine root production over the growing season to the mean standing biomass of fine roots [53]. Mean fine root biomass was estimated as the average of fine root biomass on April, May, June, July, August and September 2010.

### Litterfall and Floor Mass

Litterfall samples in each plot were collected monthly from April to October 2010 using three randomly located litter traps. Each trap (50 cm×50 cm) was made up of 1 mm mesh opening nylon netting suspended from a PVC tube square and held 5 cm above the ground by four PVC tubes. Floor mass was measured on five 50 cm×50 cm subplots randomly located in each plot every month. All plant litter samples were washed and oven-dried to constant mass at 65°C for determination of mass and chemical analysis.

### Decomposition of Leaf Litter

Decomposition rate of leaf litter was determined using the litterbag method [54]. Recently fallen leaf litter was collected from each life form. Three grams of air-dried leaf litter was placed in a 1 mm mesh opening nylon bag with a dimension of 10 cm×15 cm. We placed a total of 264 bags in six plots in June 2009. Four litterbags were collected each time at each plot after1, 2, 3, 4, 10, 11, 12, 13, 14, 15, 16 months. Collected leaf litter was oven-dried to a constant mass at 65°C. We used the following exponential function: Y_t_ = Y_0_×e^−*k*t^ [55] to determine the decay constant (*k*) and the average rate of leaf litter loss.

At the onset of the decomposition experiments, we also determined total C and N of leaf litter. Total C was determined by the standard method of wet-combustion, and total N by semi-micro Kjeldahl method [56]. The C and N contents, and the C:N ratios of the leaf litter in perennial grasses and shrubs are 43.0%, 0.9%, and 47.8, and 34.5%, 2.0% and 17.2, respectively.

### Soil Microbial Properties

Soil microbial biomass carbon (SMBC) was estimated using the fumigation-extraction method [57]. Briefly, the fresh soil samples after adjusting to approximate 60% of water holding capacity were incubated for one week in dark at 25°C. Then 20 g (dry weight equivalent) of fumigated with CH_3_Cl for 24 hours and non-fumigated soil samples were extracted with 0.5 M K_2_SO_4_. The extracts were filtered through 0.45-µm filters and analyzed for extractable organic carbon by dichromate digestion as described by Lovell et al. [31]. SMBC was calculated as the difference in extractable organic carbon content between the fumigated and non-fumigated soil samples using a conversion factor (Kec) of 0.38 to correct the incomplete extractability [57].

Soil microbial activity (SMA), i.e. soil microbial respiration, was measured by determining CO_2_ evolution over a 2-week incubation period. Briefly, 20 g (dry weight equivalent) of soil sample after adjusting to approximate 60% water holding capacity was incubated for 2-week at 25°C. Respired CO_2_ was captured in 5.0 ml of 0.5 M NaOH contained in a beaker suspended inside a Mason jar [58]. The NaOH solution was immediately titrated to determine the amount of CO_2_ evolved, and SMA was expressed as mg CO_2_ kg^−1^ d^−1^.

The metabolic quotient (*q*CO_2_: soil microbial respiration to microbial biomass carbon, mg CO_2_-C g^−1^ C_mic_ h^−1^), which representing the microbial respiration per biomass unit, was calculated according to Wardle and Ghani [59].

### Statistical Analysis

Seasonal mean values used in this study were calculated from the monthly mean values, which were first averaged from all measurements in the same month. Repeated Measures ANOVA (RMANOVA) were used to examine life form and soil layer effects on soil water content, soil organic content, fine root biomass, necromass, and also life form effects on aboveground litterfall, floor mass, soil microbial biomass carbon, soil microbial activity and *q*CO_2_ over the growing season in 2010. Two-way ANOVA was used to examine life form, soil layer and their interaction on fine root production and fine root turnover rate. Significant differences among means of measurements were determined by t test at *p* = 0.05. All the statistical analyses were performed with SPSS 11.5 software (SPSS, Chicago, IL, USA).
